# Supplementary material for: Demonstration of dual Shapiro steps in small Josephson junctions
Source: Nat Commun. 2024 Oct 9;15:8726. doi: 10.1038/s41467-024-53011-z (PMC11461542; doi:10.1038/s41467-024-53011-z)
Supplement: Supplementary file 1 — Supplementary Information [file 41467_2024_53011_MOESM1_ESM.pdf]

Supplementary Information

Fabian Kaap,[1,*] Christoph Kissling,[1] Victor Gaydamachenko,[1] Lukas Grünhaupt,[1] and Sergey Lotkhov[1]

[1] *Physikalisch-Technische Bundesanstalt, Bundesallee 100, 38116 Braunschweig, Germany*

In this supplementary we provide further information on the experimental setup, the fabrication and characterization of the device, additional measurements complementing the measurements shown in the main text and further description on the numerical model.

Experimental setup

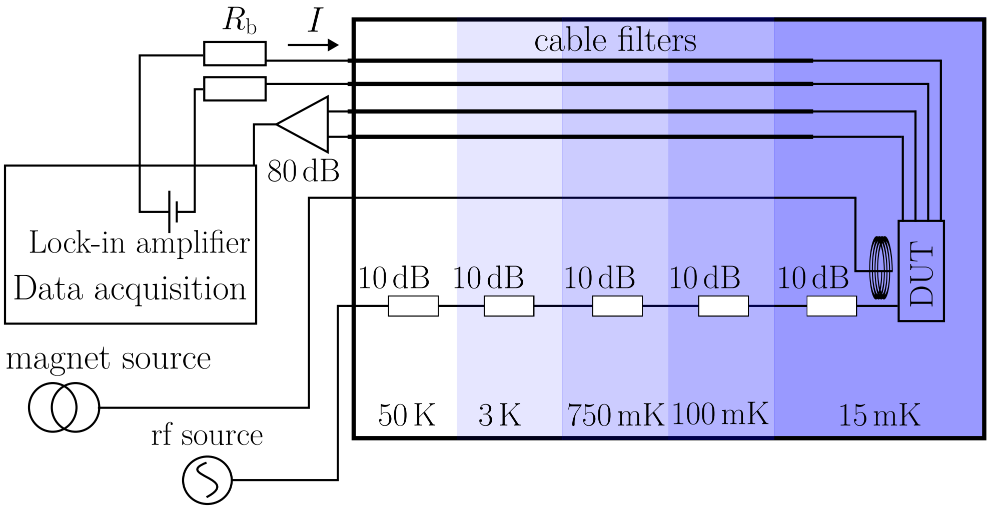

Supplementary Fig. 1. Schematics of the experimental setup. Different shades of blue indicate the temperature stages within the cryostat. See the text for detailed description of the used cabling and measuring schemes.

Supplementary Fig. 1 depicts the experimental setup. The measurements were performed in an Oxford Instruments Triton 400 dilution refrigerator. The DUT is mounted in a rf-tight copper box, which has an integrated coil to apply the magnetic flux to the SQUID and thus, control $E_{\mathrm{J,eff}}$. The dc-lines from the sample box are connected to 3 m long Thermocoax^{TM} cable filters 1, reaching from the mixing chamber stage (~ 15 mK) to the breakout connectors at the top of the cryostat. The IV-curves shown in Fig. 2 and Fig. 3(a) of the main text are measured by using CompactDAQ (cDAQ) converters from National Instruments. By applying dc-voltage V_b to two biasing resistors $R_b = 1\,\mathrm{G\Omega}$, a current $I = (V_b - U)/(2R_b + R_{\mathrm{add}})$ flows through the sample. R_{add} is the additional resistance from the cabling, the cable filters and the on-chip resistors. The voltage drop U over the DUT is amplified by the low-noise voltage amplifier Femto DLPVA-100-F-D by a factor of 10^4 (80 dB) and then digitalized using the cDAQ. For the measurements of differential resistance R_{diff} shown in Fig. 3(b-c) and Fig. 4(c-f) we use a Lock-in amplifier SR860 from Stanford Research Systems, where biasing resistors with 100 MΩ, the modulation frequency of 13.97 Hz and an amplitude of 1 mV are used. The sinusoidal drives are provided by an Anapico APMS40G rf-generator and the arbitrary waveforms are generated by a Tektronix AWG7102. The rf-signals are attenuated by 10 dB at each temperature stage of the cryostat, resulting in a total attenuation of 50 dB, neglecting additional losses from cables and connectors. Measurements at room temperature yield an upper bound for the frequency dependent losses in the coaxial cables between room temperature electronics and rf input of the chip holder of $\lesssim 1.7$ dB at 1 GHz and $\lesssim 4.2$ dB at 6 GHz. To take the data presented in Fig. 4(e) the 10 dB attenuator at the 100 mK was removed, since the output voltage of the AWG was limited to 1 V. The current for the flux coil is provided by either an Agilent B2901A SMU or by using an additional cDAQ channel.

Equivalent circuit

To visualize the individual elements of the circuit Supplementary Fig. 2 shows the equivalent circuit of the sample shown in Fig. 1(c) of the main text.

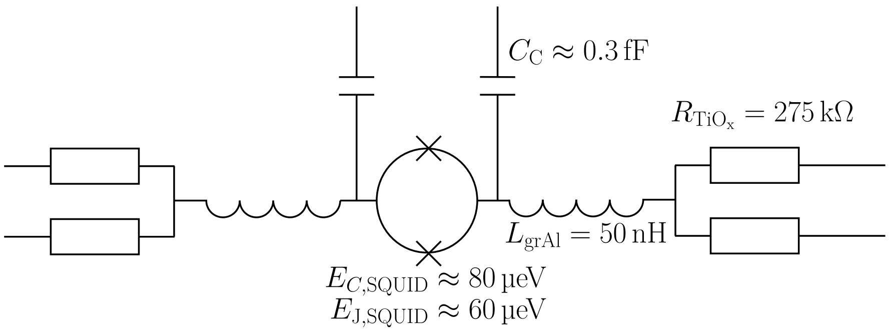

Supplementary Fig. 2. Equivalent circuit of the measured chip.

Fabrication techniques

The granular aluminium and the titanium oxide layers were fabricated in the same electron beam evaporation system. For the grAl films, Al was evaporated with an evaporation rate of $2\,\mathrm{Ås}^{-1}$, while the wafer was placed in a chamber with an oxygen pressure of $p_{\mathrm{O_2}} \approx 1.4 \times 10^{-5}\,\mathrm{mbar}$. The same procedure was used for the $\mathrm{TiO_x}$, where titanium was evaporated at a rate of $2\,\mathrm{Ås}^{-1}$ and the oxygen pressure was set to $p_{\mathrm{O_2}} \approx 3.0 \times 10^{-6}\,\mathrm{mbar}$. To ensure good electrical contact between the $\mathrm{TiO_x}$ and the following grAl and Al-layers, we sealed the end of the resistors with AuPd patches, which are less prone to oxidation in between the two fabrication steps.

Characterization of the SQUID

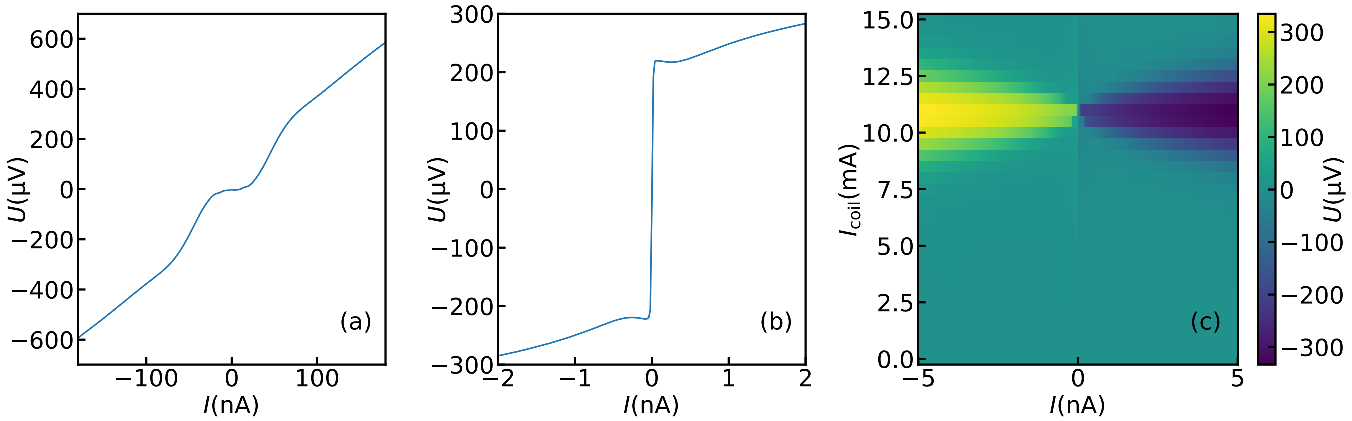

Supplementary Fig. 3. Characterization measurements: (a) Measured IV-curve for $\Phi = n\,\Phi_0$. From the critical current $I_c \approx 30\,\mathrm{nA}$ a Josephson energy $E_{\mathrm{J}} \approx 60\,\mathrm{\mu eV}$ can be estimated. (b) IV-curve at $\Phi = (n+1/2)\,\Phi_0$. We estimate the charging energy $E_C \approx 80\,\mathrm{\mu eV}$. (c) IV-curves for different coil currents I_{coil}. E_{J} is maximally suppressed at $I_{\mathrm{coil}} \approx 11\,\mathrm{mA}$.

Supplementary Fig. 3(a) shows the IV-curves for $\Phi = n\,\Phi_0$, where the critical current $I_{\mathrm{c}} \approx 30\,\mathrm{nA}$, defined as the onset of the resistive branch, is maximized. The unsuppressed Josephson energy is thus $E_{\mathrm{J}} = \frac{\Phi_0 I_{\mathrm{c}}}{2\pi} \approx 60\,\mathrm{\mu eV}$. From the IV-curve in Supplementary Fig. 3(b) for $\Phi \to (n+1/2)\,\Phi_0$, where the Coulomb blockade is maximized, we can

estimate the charging energy $E_C = 80\,\mu\text{eV}$. In Supplementary Fig. 3(c) the IV-curves as a function of the coil current I_{coil} are shown. At $I_{\text{coil}} \approx 11\,\text{mA}$ the flux value $\Phi = \Phi_0/2$ is reached. From measurements up to $I_{\text{coil}} = 35\,\text{mA}$ we extract a current-to-flux coefficient of $\sim 22.5\,\text{mA}\Phi_0^{-1}$.

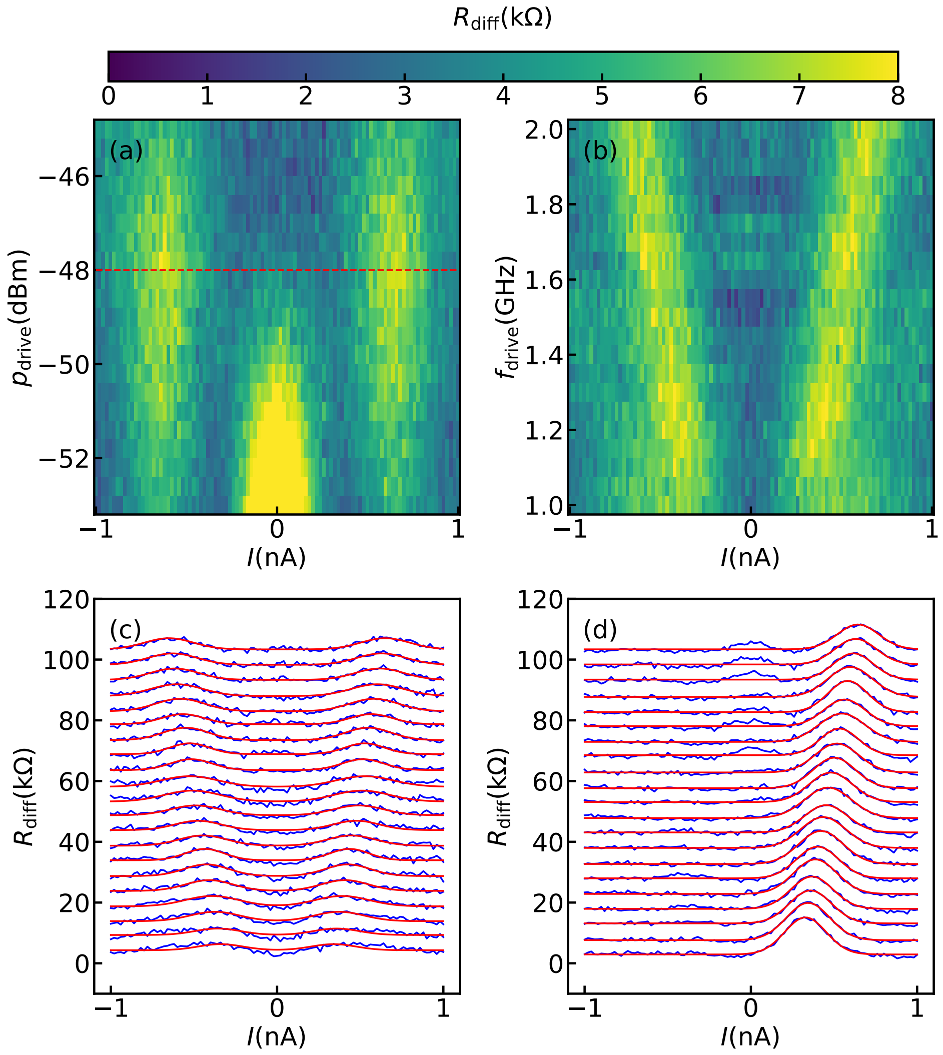

Supplementary Fig. 4. Detailed plots for Fig. 4 of the main text. (a) R_{diff} for different drive powers p_{drive}. To find the best comparison with the measurement using a pulsed drive, we chose $p_{\text{drive}} = -48\,\text{dBm}$ (indicated with red line), at which the first dual Shapiro step is maximized. (b) R_{diff} for different frequencies f_{drive} of the sinusoidal drive. (c) Gaussian fits to the data shown in (b). For visualization purposes an offset of $3\,\text{k}\Omega$ is added to each line. (d) Gaussian fits to the data shown in Fig. 4(f) of the main paper. An offset of $5\,\text{k}\Omega$ is added to each line for clarity. The data shown in the inset of Fig. 4(f) are extracted from this measurement.

Quantifying dual Shapiro steps

The differential resistance R_{diff} for values between $-52.8\,\text{dBm} \leq p_{\text{drive}} \leq -44.2\,\text{dBm}$ is shown in Supplementary Fig. 4(a). The red line at $-48\,\text{dBm}$ indicates, where the measured peak $R_{\text{diff,peak}}$ of the first dual Shapiro step is maximized and is used for comparison in Fig. 4(b) in the main text. To quantify the difference between the sinusoidal and the pulsed drive, we measured, analogue to Fig. 4(d) of the main text, the differential resistance for sinusoidal drive frequencies $1\,\text{GHz} \leq f_{\text{drive}} \leq 2\,\text{GHz}$ at $p_{\text{drive}} = -48\,\text{dBm}$, as shown in Supplementary Fig. 4(b). The peak values $R_{\text{diff,peak}}$ shown in the inset of Fig. 4(d) of the paper are extracted by fitting Gaussians to the measurements of Fig. 4(b) and Fig. 4(d) of the paper. In Supplementary Fig. 4(c) and Supplementary Fig. 4(d) the fits for a set of frequencies are shown. Supporting the assumption that the smearing of the dual Shapiro steps occurs due to thermal

broadening, fitting a Gaussian leads to good agreement with the measurements. The error bars in the inset of Fig. 4(f) of the main text are given by the error on the current of the dual Shapiro step.

Numerical analysis of different drives

To evaluate the influence of different drives on the dual Shapiro steps, we make use of the duality principle. For simplicity, we assume a sinusoidal shape of the lowest Bloch band and neglect all higher energy bands. With this single band approximation the dynamics of the quasicharge q is described by

$$L\ddot{q} + R\dot{q} + V_{\mathrm{c}} \sin\left(\frac{\pi}{e} q\right) = V_{\mathrm{tot}} = V_{\mathrm{dc}} + V_{\mathrm{ac}}(t). \tag{S.1}$$

With $\tau = t\frac{\pi V_{\mathrm{c}}}{eR}$, $\beta = \frac{\pi V_{\mathrm{c}} L}{eR^2}$, $\tilde{q} = \frac{\pi}{e} q$ and $\alpha_{\mathrm{dc,ac}} = V_{\mathrm{dc,ac}}/V_{\mathrm{c}}$ the equation (1) simplifies to

$$\beta \frac{d^2\tilde{q}}{d\tau^2} + \frac{d\tilde{q}}{d\tau} + \sin\tilde{q} = \alpha_{\mathrm{dc}} + \alpha_{\mathrm{ac}} f(\tau) \tag{S.2}$$

and can be solved using a fourth-order Runge-Kutta method [2]. Supplementary Fig. 5 shows the calculated IV-curves for different drive powers for a pulsed (a) and a sinusoidal (b) driving signal. For pulsed driving, the Coulomb blockade (colored region indicated by "0") gradually vanishes with increasing power and the first dual Shapiro step (colored region indicated by "+1") grows up to the point $\alpha_{\mathrm{ac}} \approx 10$, where the second dual Shapiro steps starts appearing. The negative dual Shapiro steps never appear in this calculation due to the sign of the current pulse. Changing the sign of the pulse would lead to a similar pattern of steps at negative currents. The measured power dependence shown in Fig. 4(d) of the main paper qualitatively supports this calculation. For a sinusoidal drive shown in Supplementary Fig. 5(b), increasing the power leads to a suppression of the Coulomb blockade and an appearance of both positive and negative steps symmetrically until at $\alpha_{\mathrm{ac}} = 2.5$ the CB completely vanishes. Further increasing the power leads to a reappearing of the Coulomb blockade and a pronounced second dual Shapiro step. This pattern is matching the measurement shown in Fig. 3(c) in the main text.

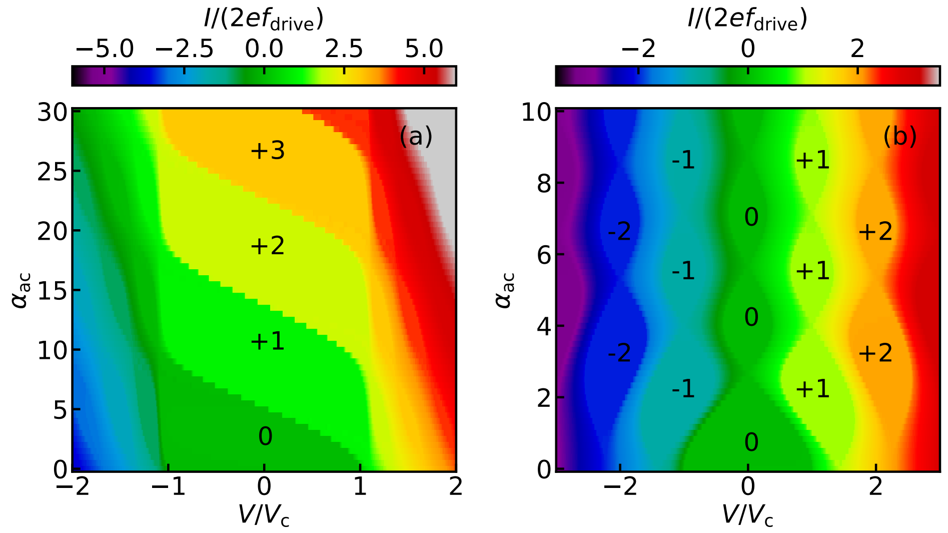

Supplementary Fig. 5. Simulated IV-curves for different drive powers obtained by solving Eq. (2) using the fourth-order Runge-Kutta method. (a) Pulsed drive with pulse duration $t_p = 0.05 \cdot f_{\mathrm{drive}}^{-1}$. The region 0 indicates the Coulomb blockade, while the numbers and sign indicate the order and sign of the dual Shapiro step. For a pulsed drive dual Shapiro steps only occur for one current direction. (b) Sinusoidal drive leads to a symmetric power dependence of the IV-curves and appearance of positive and negative dual Shapiro steps as indicated in the plot.

Temperature dependence of the Coulomb blockade

Supplementary Figure 6(a) shows the temperature dependence of the IV-curves at an external flux of $\Phi \approx 0.3\Phi_0$. One can see that the Coulomb blockade is shrinking with increasing temperature. The extracted voltage of the Coulomb blockade V_c is shown in Supplementary Fig. 6(b). The Coulomb blockade shows a small plateau up to 40 mK and then starts to shrink with increasing temperature. This indicates that without rf-drive the electron temperature is close to the fridge temperature. To get an estimate of the electron temperature during the irradiation of the rf-drive, one can compare the IV-curve of Fig. 3(a) to the one of Supplementary Fig. 6. We thus expect the electron temperature during the rf-drive to be around 200 mK to 250 mK.

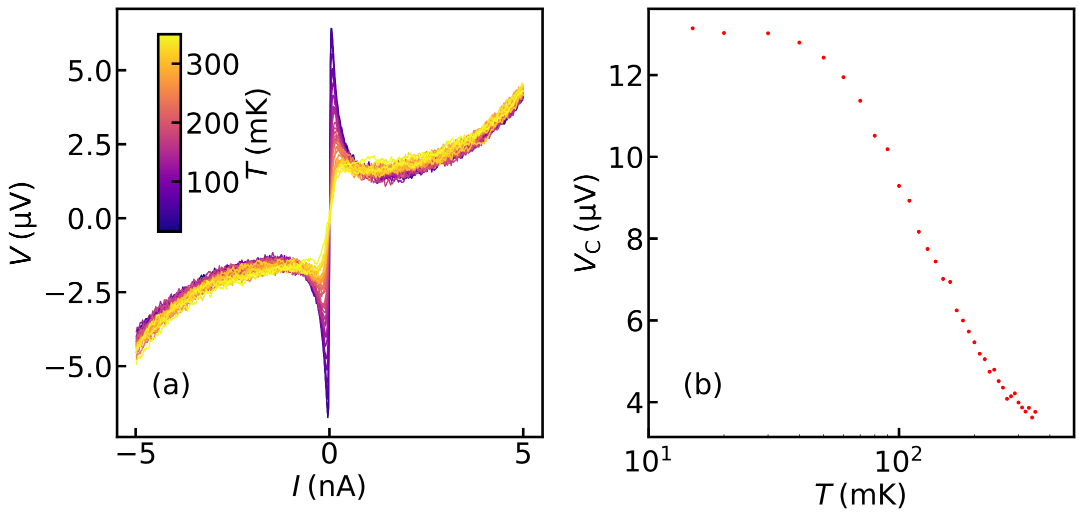

Supplementary Fig. 6. (a) IV-curves of the sample at $\Phi \approx 0.3\Phi_0$ at different temperatures (indicated by the color). (b) Extracted Coulomb blockade V_c as a function of the temperature.

Choice of flux point

To investigate the effect of the flux bias point on the dual Shapiro steps the current of the coil generating the magnetic field was varied, while the rf drive was fixed at a power $p_{\mathrm{drive}} = -48\,\mathrm{dBm}$ and frequency $f_{\mathrm{drive}} = 3.2\,\mathrm{GHz}$ and the IV-curves were measured. To find a good trade-off between the width of the dual Shapiro step and the Landau-Zener tunneling branch, the differential resistance normalized to the average of the normal resistance $\bar{R}_{\mathrm{diff}}$ was plotted in Supplementary Fig. 7 (a). It can be seen, that a small coil current I_{coil}, the dual Shapiro steps can not be seen due to the noise of the measurement (red). Increasing I_{coil} makes the dual Shapiro step visible (orange). Further increasing I_{coil} increases the width of dual Shapiro step but at the same time the Landau-Zener tunneling, such that the normalized R_{diff} shows a less pronounces dual Shapiro step (magenta). The IV-curves of the three coil currents indictated by the colored lines are shown in Supplementary Fig. 7(b). The orange curve shows the clearest dual Shapiro step, while the red curve is dominated by the noise of the measurement and the magenta curve is showing a less pronounced step when comparing it to the voltage increase due to Landau-Zener tunneling.

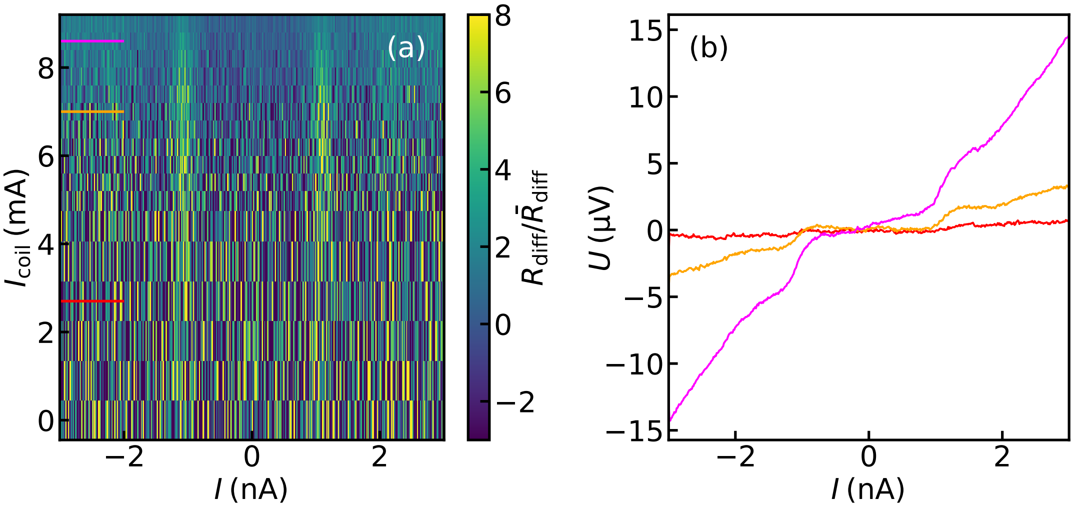

Supplementary Fig. 7. (a) Differential resistance extracted from measuring the IV-curves and normalized to the average differential resistance to take the Landau-Zener tunneling into account. (b) IV-curves of the three colored lines in (a).

SUPPLEMENTARY REFERENCES

* fabian.kaap@ptb.de

[1] A. B. Zorin, The thermocoax cable as the microwave frequency filter for single electron circuits, Review of Scientific Instruments **66**, 4296 (1995).

[2] S. Maggi, Step width enhancement in a pulse-driven josephson junction, Journal of Applied Physics **79**, 7860 (1996).
